# Disrupted Spontaneous Neural Activity and Its Interaction With Pain and Emotion in Temporomandibular Disorders

**DOI:** 10.3389/fnins.2022.941244

**Published:** 2022-08-25

**Authors:** Xiao-Fei Chen, Ping He, Kuang-Hui Xu, Yi-Han Jin, Yong Chen, Bin Wang, Xu Hu, Le Qi, Ming-Wei Wang, Jie Li

**Affiliations:** ^1^Department of Radiology, The Affiliated Hospital of Hangzhou Normal University, Hangzhou, China; ^2^Department of Orthodontics, Hangzhou Stomatological Hospital, Hangzhou, China; ^3^Department of Oral and Maxillofacial Surgery, The Affiliated Hospital of Hangzhou Normal University, Hangzhou, China; ^4^Department of Cardiology, The Affiliated Hospital of Hangzhou Normal University, Hangzhou, China

**Keywords:** temporomandibular disorders, chronic pain, emotions, resting-state fMRI, amplitude of low-frequency fluctuation, functional connectivity

## Abstract

**Background and Purpose:**

Temporomandibular disorders (TMD), especially pain-related TMD, are closely related to social and psychological factors. We aimed to measure changes in spontaneous brain activity and its related functional connectivity (FC), as well as FC characteristics within the mood-regulating circuits (MRC) in TMD patients by resting-state functional magnetic resonance imaging (RS-fMRI), and to analyze the relationship between these parameters and emotional symptoms.

**Materials and Methods:**

Twenty-one adult TMD patients and thirty demographically matched healthy controls (HCs) underwent clinical scale evaluation and RS-fMRI scanning. After processing RS-fMRI data, the values of the amplitude of low-frequency fluctuation (ALFF) between the two groups were compared. Regions with abnormal ALFF values were selected as areas of interest (ROIs) to compare the differences of whole-brain seed-based FC between groups. The FCs between regions within MRC were also analyzed and compared. In addition, the relationships between RS-fMRI characteristics and pain and mood were explored by correlation and mediation analyses.

**Results:**

Compared with HCs, TMD patients showed increased ALFF in the right parahippocampal gyrus (PHG), the right supplementary motor area, and the bilateral precentral gyrus, with decreased ALFF in the right cerebelum_crus2. Patients showed enhanced right PHG-related FC in the vermis and posterior cingulate cortex, orbitofrontal cortex (OFC)-related FC in the striatal-frontal regions, while decreased dorsolateral prefrontal cortex-related FC in the amygdala. In TMD patients, ALFF values in the right PHG and FC values between the right PHG and the vermis were positively correlated with depressive symptoms. Abnormal FCs in the left striatal-orbitofrontal pathway were correlated with pain and depressive symptoms. More importantly, mediation analysis revealed that chronic pain mediates the relationship between FC of right PHG with vermis and depressive symptoms, and abnormal FC in the left striatal-orbitofrontal pathway can mediate the association between pain and depressive symptoms.

**Conclusion:**

TMD patients have dysregulated spontaneous activity and FC in the default mode network, sensorimotor network and pain-related regions, as well as dysfunction of the fronto-striatal-limbic circuits. The development of negative emotions in TMD may be related to the dysfunction of components within the reward system (especially hippocampus complex, OFC, striatum) due to chronic pain.

## Introduction

Temporomandibular disorders (TMD) are a common clinical oral disease characterized by oral-facial pain, joint sounds, and mandibular movement disorders, but the pathogenesis remains unclearly. However, it is thought to be related to psychological, occlusal factors, joint and muscle factors (Scrivani et al., 2008; Schiffman et al., 2014). The diagnostic criteria for TMD require a comprehensive assessment of psycho-psychological status in addition to the evaluation of physical symptoms (Schiffman et al., 2014). Anxiety and depression are highly co-occurring within the TMD population, especially in patients with myofascial pain (Reis et al., 2022). Relieving stress and depression can effectively improve TMD-related pain (Gauer and Semidey, 2015). Recent studies have suggested that TMD may have similar pathophysiological features related to the central nervous system as chronic fatigue syndrome and chronic lower back pain (Scrivani et al., 2008; Woolf, 2011). Therefore, exploring the characteristics of neuronal activity can promote the understanding of the pathophysiological mechanism of TMD, and recognizing the impact of emotional symptoms on TMD may have implications for treatment options and clinical outcomes.

Functional magnetic resonance imaging (fMRI) is a powerful non-invasive tool for assessing brain function, which can reflect neuronal activity by detecting relative changes in local deoxyhemoglobin content. The experimental paradigm of fMRI includes task-state fMRI and resting-state fMRI (RS-fMRI). Previous task-fMRI studies have revealed decreased motor and cognitive functions in TMD patients and associated with abnormal activation of brain regions implicated in attention, emotional processes, motor planning, and performance, particularly the prefrontal cortex, the parietal cortex, the cingulate cortex, and the amygdala (Nebel et al., 2010; Weissman-Fogel et al., 2011; Ichesco et al., 2012). RS-fMRI can detect spontaneous neural activity without specific tasks with a high spatial resolution (Fox and Raichle, 2007). Analysis methods of RS-fMRI data can be divided into two categories: functional segregation and functional integration (Lv et al., 2019). Functional separation is the analysis of local spontaneous brain activity, and its commonly used analysis indicators include the regional homogeneity (ReHo) (Zang et al., 2004), the amplitude of low-frequency fluctuation (ALFF) (Zang et al., 2007), the fractional ALFF (fALFF) (Zou et al., 2008), etc. Functional integration focuses on the relationships or connections between different brain regions, and functional connectivity (FC) is the most commonly used analysis method (Biswal et al., 1995). The existing RS-fMRI studies on TMD mainly use the FC method based on the region of interest (ROI) to observe the correlation between brain regions. Disruption of FCs between brain regions in the corticostriatal networks, the salience network, and the default mode network were found in TMD patients, which may underlie the deficits in motor control, pain processing, and cognition in TMD (Ichesco et al., 2012; Kucyi et al., 2014; He et al., 2018; Zhang et al., 2018). However, most of the above studies are hypothesis-driven analyses based on the ROIs selected by researchers who propose hypotheses. Through the fALFF method of functional separation analysis, He et al. (2014) found that stable splint therapy could promote the recovery of local abnormal activity in the prefrontal cortical areas of TMD patients. Therefore, it is of great significance to combine functional separation and functional integration to reveal the characteristics of abnormal activities in local brain regions and their FC patterns to the whole brain.

The neurophysiological basis of emotion generation and perception involves a wide range of cortical and subcortical regions (e.g., striatum, hippocampal complex, amygdala, thalamus), which is also known as the fronto-striatal-limbic mood regulation circuit (MRC) (Anand et al., 2005). Neuroimaging studies have found that most brain regions with abnormal neural activity in TMD patients are located in or related to MRC (Moayedi et al., 2011, 2012; Gerstner et al., 2012; Ichesco et al., 2012; He et al., 2018; Lim et al., 2021). However, their relationship with emotional symptoms and the role between pain and emotion remains unclear. Based on previous research and theoretical results, we utilized the ALFF method for data-driven analysis and combined it with FC analysis to explore changes in whole-brain spontaneous neural activity and FC in patients with TMD. In addition, we also focus on the FC characteristics between regions within the MRC. The two approaches define the brain functional characteristics of TMD patients from different perspectives and present a complementary relationship. We hypothesized that there would be aberrant brain function in emotion and pain-related processing regions and disrupted FC within MRC in TMD patients, and the partly abnormalities would be associated with emotional symptoms. In particular, chronic oral-facial pain may mediate this correlation.

## Materials and Methods

### Participants

Twenty-four patients with TMD and thirty age and sex-matched HCs were recruited from the orthodontic clinic and physical examination center of the Affiliated Hospital of Hangzhou Normal University and Hangzhou dental Hospital. This study was approved by the Ethics Committee of the Affiliated Hospital of Hangzhou Normal University. All subjects provided written informed consent before participating.

### Inclusion and Exclusion Criteria for Participants

All patients with TMD were carefully examined by two dentists with experience in orofacial pain and met the evidence-based Diagnostic Criteria for pain-related TMD (DC/TMD; Group I) (Schiffman et al., 2014). The inclusion criteria included: (1) age 18-55 years; (2) full permanent dentition; (3) presence of pain in the face, jaw, or temples under the close-mouth state; (4) presence of pain symptoms for greater than 3 months. The exclusion criteria included a history of orthodontic or TMD-related treatment, chewing side preference, chronic maxillofacial or other chronic pain unrelated to TMD (e.g., migraine, irritable bowel syndrome, or fibromyalgia), a history or current diagnosis of psychosocial disorders (e.g., major depression, psychosis, or drug and alcohol abuse as defined by the DSM-IV-TR criteria), a history of brain trauma or craniocerebral surgery, pregnancy or lactation, and MR contraindications. The inclusion criteria for the HCs were full permanent dentition, no presence of any pain, and no symptoms or signs of TMD, and the exclusion criteria were the same as those of the TMD patients. All participants were right-handed according to the Edinburgh Hand-Handling Scale.

### Questionnaire Scale Assessment

Prior to MRI examination, the clinical data of TMD patients were collected, including the course of the disease and visual analog scale (VAS) (Huskisson, 1974). The VAS was used to measure pain intensity under a mouth-closed state (range: 0–10; 0 refers to no pain and 10 refers to the worst pain imaginable). All subjects completed 17-items of the Hamilton Depression Scale (HAMD-17) (Hamilton, 1960) and 14-items of the Hamilton Anxiety Scale (HAMA-14) (Thompson, 2015) under the guidance of two psychiatrists. These two questionnaires are the most commonly used scales in the clinical assessment of depression and anxiety. Scores above seven indicate depression or anxiety.

### Magnetic Resonance Imaging Data Acquisition

Brain imaging data were collected on a GE Discovery-750 3.0T MRI (GE Discovery MR-750, Milwaukee, WI). Subjects were required to lie on the MRI scanning bed, and sponge cushions were used to fix both sides of the head to minimize head movement. During the image acquisition process, the participants need to remain awake, relax with their eyes closed, breathe smoothly and keep their heads motionless as much as possible. The scanning sequences included the RS-fMRI image and the whole-brain three-dimensional high-resolution T_1_-weighted image (3D T_1_WI). RS-fMRI data were obtained by the blood oxygen level-dependent (BOLD) gradient echo-echo planar imaging sequence. The parameters are repetition time (TR) = 2,000 ms, echo time (TE) = 30 ms, flip angle (FA) = 90^°^, field of view (FOV) = 192 mm × 192 mm, acquisition matrix = 64 × 64, layer thickness = 4 mm, interval = 0 mm. The scan range includes the whole brain, and the scan time is 8 min in total. 3D T_1_WI data were acquired using the three-dimensional spoiled gradient recalled-echo (SPGR) sequence, and the parameters are TR = 8.16 ms, TE = 3.18 ms, FA = 8^°^, FOV = 256 mm × 256 mm, acquisition matrix = 256 × 256, layer thickness = l mm, 176 layers. In addition, T_1_WI and T_2_ fluid-attenuated inversion recovery sequences were also collected to exclude abnormal anatomical structures and organic brain diseases.

### Magnetic Resonance Imaging Data Preprocessing

Based on the Matlab R2019 platform,^1^ Data Processing and Analysis for (Resting-State) Brain Imaging (DPABI^2^) software was used for data preprocessing in Statistical Parametric Mapping version 12 (SPM 12^3^) operating environment. The pre-processing steps are as follows: MRI data of all subjects were converted from DICOM format to NIFTI format by MRI CONVERT software^4^; removed the first 10 volumes to achieve signal balance and participant adaptation to the scan; time level correction: the data at each time point was corrected for the difference of collection time points to eliminate the interval scanning time difference; head movement correction: removed the data of subjects whose head movement in x, y and z axis is more than 3mm or rotation angle is more than 3°; space standardization: functional images were matched with structural images by DARTEL and registered into Montreal Neurological Institute (MNI) space, and resampled to 3 × 3 × 3mm^3^ voxel size; smoothed with a 6mm full-width at half-maximum (FWHM) Gaussian kernel to improve the signal-to-noise ratio of the image and to remove the linear drift of the data; the whole-brain signal, white matter signal, cerebrospinal fluid signal and head movement parameters were regressed as covariables.

### Amplitude of Low-Frequency Fluctuation Calculation

The ALFF values were performed and calculated by DPABI software. The fast Fourier transform was used to transform the time series of a given voxel into a frequency domain. The ALFF values were calculated by taking the square root of the power spectrum and averaging it across the low-frequency band (0.01∼0.08 Hz). The ALFF values of each voxel were divided by the global mean ALFF value to standardize data across subjects.

### Functional Connectivity Calculation

Data-driven FC analyses based on ROIs to whole-brain voxel-wise were used to calculate the differences in connection patterns between ROIs and whole-brain between the two groups. Based on the results of ALFF analyses, the abnormal regions were chosen as the ROIs (spherical regions with a radius of 5 mm centered on the peak coordinates), including the right parahippocampal gyrus (PHG), the right superior frontal gyrus, the right cerebelum_crus2, and the bilateral precentral gyrus (GRF correction, *P* < 0.05 for the cluster threshold and *P* < 0.001 for the voxel threshold, two-tailed). The mean time series of these ROIs were extracted. Then the correlation coefficient (*r*-value) between them and the time series of each voxel in the whole brain was calculated to generate correlation graphs. Finally, Fisher’s *r* to *z* transformation was carried out to improve the normality of the correlation coefficient.

ROI-wise FC analyses based on prior assumptions were used to calculate the difference of FCs in the MRC between the two groups. According to previous studies, the regions involved in emotion and pain regulation are mainly located in the dorsal and ventral pathways, including the dorsolateral prefrontal cortex (DLPFC), the ventral lateral prefrontal cortex (VLPFC), the medial prefrontal cortex (mPFC), the anterior cingulate cortex (ACC), the orbitofrontal cortex (OFC), the amygdala and the ventral striatum (Phillips et al., 2008; Kupfer et al., 2012). Therefore, we defined the above seven brain regions of bilateral cerebral hemispheres as ROI, that is, a ball with a diameter of 5 mm was drawn with the coordinate point as the central point [MNI (x, y, z): DLPFC (left: −37, 46, 36; right: 46, 28, 31), VLPFC (left: −24, 41, 14; right: 38, 42, 14), mPFC (left: −10, 41, 6; right: 8, 42, 5), ACC (left: −5, 35, 14; right: 7, 37, 15), OFC (left: −24, 52, −12; right: 24, 52, −11), amygdala (left: −24, −1, −17; right: 26, −1, −17), ventral striatum (left: −18, 16, −2; right: 18, 20, −6)]. The above coordinates were selected according to the Yeo atlas (Yeo et al., 2011). The correlation coefficients (*r*-values) between pairs of time series within these fourteen ROIs were calculated to generate the correlation graph, and then Fisher’s *r* to *z* transformation was performed.

### Statistical Analysis

Demographic and clinical data were analyzed using SPSS software Version 25 (SPSS, Chicago, Illinois, United States). Two-sample *t*-tests were used to explore the ALFF and the FC (ROI to whole-brain FC value and ROI to ROI FC value) differences between the two groups. Age, gender, and head movement parameters were used as covariates in these comparisons (GRF correction, *P* < 0.05 for the cluster threshold and *P* < 0.001 for the voxel threshold, two-tailed). In the TMD group, the values of ALFF and FC of a spherical region with a radius of 5mm centered on the peak coordinates were extracted. Partial correlation analyses were performed between altered ALFF or FC values and pain/mood symptoms. If there was a significant correlation, a tentative bootstrapped mediation analysis was conducted using the PROCESS macro in SPSS (Hayes, 2013) to explore the relationship between abnormal ALFF in a specific brain region or abnormal FC between brain regions, pain degree, and emotional symptoms. The significance was estimated using a bias-corrected bootstrapping method with 5,000 iterations. In the correlation and mediation analysis, gender and age were taken as covariables, and *P* < 0.05 was considered statistically significant.

## Results

### Demographic and Clinical Results

Three patients were excluded, including one whose MRI image revealed intracranial lesions (white matter hyperintensity) and two people whose RS-fMRI data did not meet the processing requirements due to excessive head movement. Thus, 51 participants were recruited, including a TMD group (*n* = 21, 7 with unilateral left maxillofacial pain, 6 with unilateral right pain, 8 with bilateral pain) and HCs (*n* = 30).

Demographic details and clinical characteristics of all TMD patients and HCs were summarized in Table 1. There were no significant differences between the groups concerning sex (*t* = 3.757, *P* = 0.053) or age (*t* = −0.429, *P* = 0.669). The mean disease duration of TMD patients (± standard deviation) was 8.90 ± 5.72 months, with the VAS scores was 4.29 ± 2.00. The depression and anxiety scores of TMD patients were higher than those of HCs (*t* = 7.297, *P* < 0.001; *t* = 8.796, *P* < 0.001).

**TABLE 1:** Demographic and clinical characteristics of participants.

Variables	TMD (*n* = 21)	HCs (*n* = 30)	*t* values	*P*-values
Sex (male/female)	9/12	21/9	3.757	0.053^a^
Age (years)	27.95 ± 4.56	28.57 ± 5.33	−0.429	0.669^b^
HAMD-17	4.00 ± 2.21	0.33 ± 0.76	7.297	<0.001^b^
HAMA-14	4.81 ± 2.21	0.40 ± 0.77	8.796	<0.001^b^
VAS	4.29 ± 2.00	—	—	—
Duration (months)	8.90 ± 5.72	—	—	—

*^a^Assessed by chi-square test. ^b^Assessed by independent-sample t-test, two-tailed; Data are shown as mean ± standard deviation. HAMD-17, 17-item Hamilton Depression Rating Scale; HAMA-14, 14-item Hamilton Anxiety Rating Scale; VAS, Visual Analog Scale; TMD, temporomandibular disorder; HCs, healthy controls.*

### Amplitude of Low-Frequency Fluctuation and Data-Driven Functional Connectivity Analyses

**FIGURE 1:**
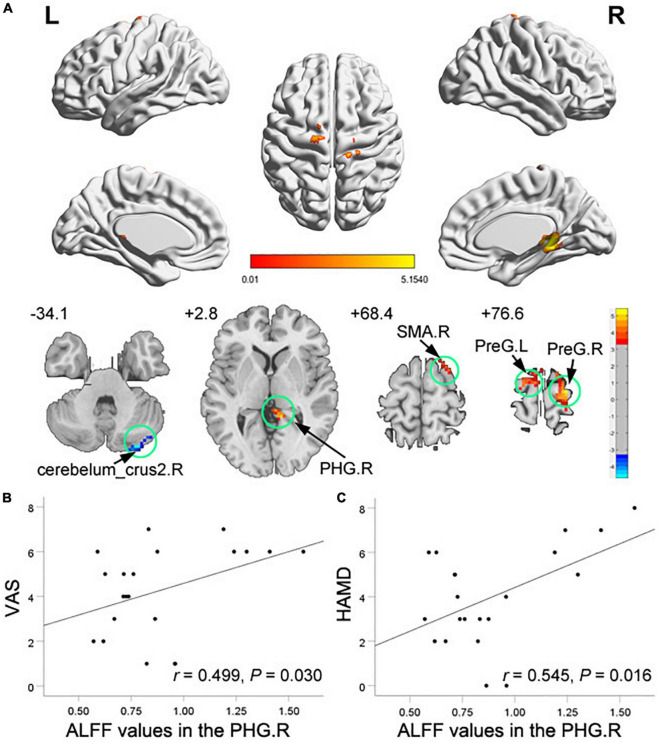
Clusters showing amplitude of low-frequency fluctuation (ALFF) changes in temporomandibular disorders (TMD) patients as compared to that in healthy controls **(A)**, and correlations of abnormal ALFF values with visual analog scale (VAS) scores **(B)** and HAMD scores **(C)**. The significance threshold was set at voxel-level *P* < 0.001 and cluster-level *P* < 0.05 (GRF corrected). Blue color denotes relatively lower ALFF values in the TMD group, red color denotes relatively higher ALFF values in the TMD group, and the color bar indicates the t-value from the two-sample *t*-test between the TMD group and HCs. There were significant positive correlations between the ALFF values of the right PHG and the VAS and the HAMD scores in TMD patients. PHG, parahippocampa gyrus; SMA, supplementary motor area; PreG, precentral gyrus; R, right; L, left.

Compared with HCs, TMD patients showed increased ALFF in the right PHG, the right supplementary motor area (SMA), and the bilateral precentral gyrus, with decreased ALFF in the right cerebelum_crus2 (Figure 1A and Table 2). In TMD patients, ALFF values in the right PHG were positively correlated with pain and depressive symptoms (*r* = 0.499, *p* = 0.030; *r* = 0.545, *p* = 0.016) (Figure 1B,C).

**TABLE 2:** Brain regions showing significant differences in amplitude of low-frequency fluctuation (ALFF) between groups.

	MNI coordinates of maximum voxel			Cluster size (voxels)	BA	Peak voxel *t*-value	Anatomical region
	*x*	*y*	*z*				
**TMD > HCs**							
	12	–39	0	36	30	5.07	right parahippocampa gyrus
	18	24	66	46	6	4.51	right supplementary motor area
	–18	–12	78	27	6	4.22	left precentral gyrus
	24	–18	78	35	6	4.67	right precentral gyrus
**TMD < HCs**							
	30	–87	–33	27		−4.70	right cerebelum_crus2

*All statistical results were thresholded at voxel-level P < 0.001 and cluster-level P < 0.05, GRF -corrected. Brain regions were described according to the Anatomical Automatic Labeling (AAL) templates. BA, brodmann area, TMD, temporomandibular disorder; HCs, healthy controls.*

**FIGURE 2:**
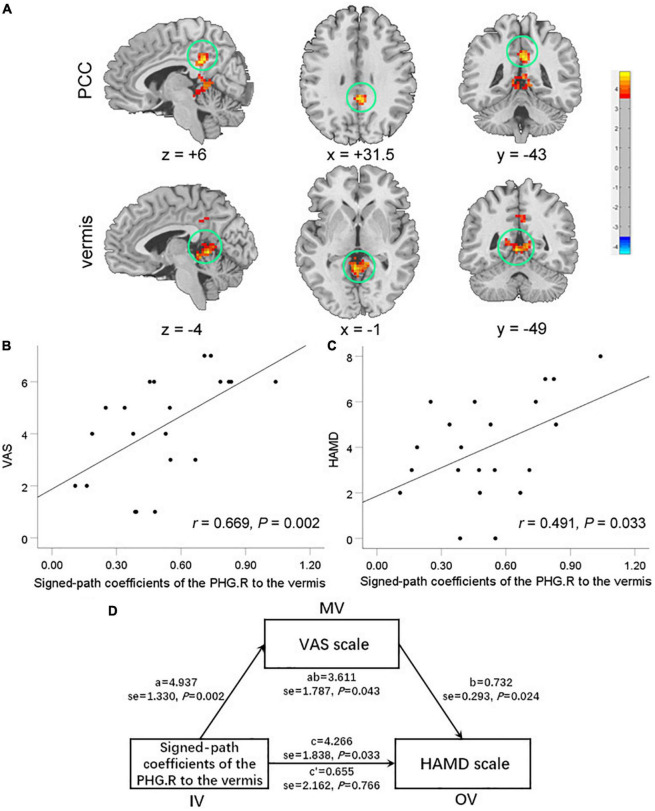
Brain regions exhibiting enhanced functional connectivity (FC) with the right PHG in temporomandibular disorders (TMD) patients compared with healthy controls **(A)**. There were significant positive correlations between the FC of the right PHG with vermis and the visual analog scale (VAS) scores **(B)** and the HAMD scores **(C)**. The oral-facial pain completely mediated the relationship between the FC of the right PHG with vermis and the HAMD scores **(D)**. PCC, posterior cingulate cortex; PHG, parahippocampa gyrus; VAS, Visual Analog Scale; HAMD, Hamilton Depression Rating Scale.

Compared to the HCs, TMD patients showed enhanced connectivity between the right PHG [the peak point coordinates was (12, −39, 0)] and the vermis [the peak point coordinates was (−3, −48, 0), the peak *t*-value was 4.824, and the number of voxels was 210], and the posterior cingulate cortex (PCC) [the peak point coordinates was (3, −42, 33), the peak *t*-value was 4.879, and the number of voxels was 90] (Figure 2A).

In TMD patients, FC values between the right PHG and the vermis were positively correlated with pain and depressive symptoms (*r* = 0.669, *P* = 0.002; *r* = 0.491, *P* = 0.033) (Figures 2B,C). The mediating analysis showed that the FC values between right PHG and vermis had a significant overall effect on depression (*P* = 0.043), and were completely mediated by oral-facial pain intensity. The statistical values of the specific mediation model were as follows: Path c, *P* = 0.033; Path a, *P* = 0.002; Path b, *P* = 0.024; Path c′, not significant; Standardization indirect effect = 3.611, 95% confidence interval: 0.594, 7.544 (Figure 2D).

### Region of Interest-Wise Functional Connectivity Analyses Within Mood-Regulating Circuits

**FIGURE 3:**
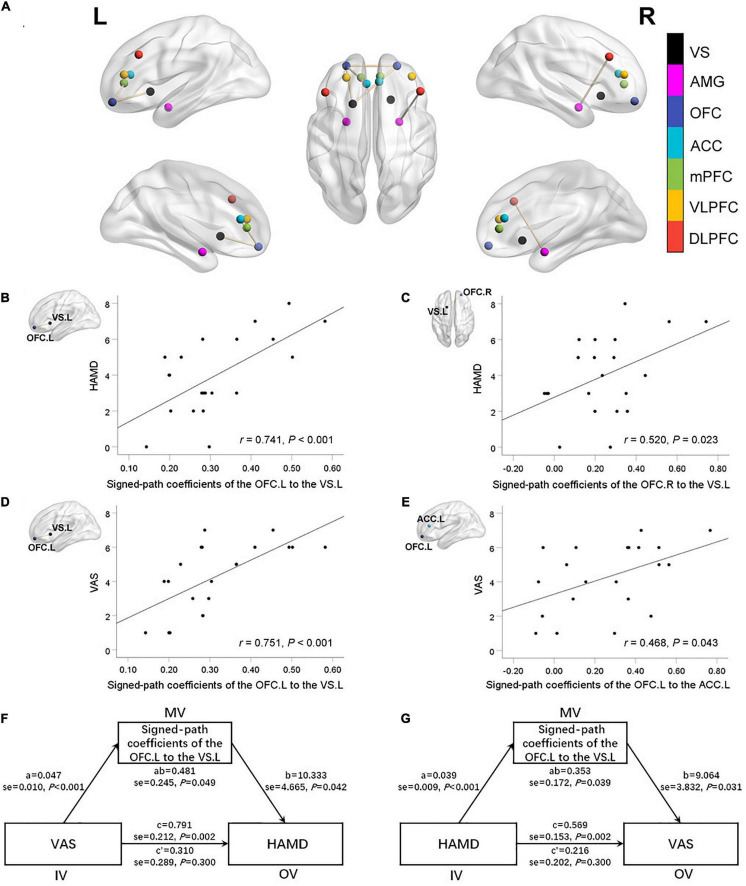
Abnormal functional connectivity (FC) between selected brain regions in mood-regulating circuits (MRC) between the two groups **(A)**. There were significant positive correlations between the FC of the left VS with left OFC **(B)**, right OFC **(C)** and the HAMD scores. There were significant positive correlations between the FC of the left OFC with left VS **(D)**, left ACC **(E)** and the VAS scores. The oral-facial pain and depressive symptoms are mutually significant overall effects. Both relationships were completely mediated by the FC between the left OFC and left VS **(F,G)**. VS, ventral striatum; AMG, amygdala; OFC, orbitofrontal cortex; ACC, anterior cingulate cortex; mPFC, medial prefrontal cortex; VLPFC, ventral lateral prefrontal cortex; DLPFC, dorsolateral prefrontal cortex; HAMD, Hamilton Depression Rating Scale; VAS, Visual Analog Scale; R, right; L, left.

Pairwise FC comparison of MRC-associated regions between the two groups showed FC between the left OFC and the left ACC (T values = 2.06), the left OFC and the left ventral striatum (T values = 3.03), the right OFC and the left OFC (T values = 2.23), the right OFC and the left ventral striatum (T values = 2.37) were increased in the TMD patients, while FC between the right DLPFC and the right amygdala was decreased (T values = −2.03) (Figure 3A).

In TMD patients, FCs between the left OFC and the left ventral striatum, the right OFC and the left ventral striatum were positively correlated with depressive symptoms (*r* = 0.741, *P* < 0.001; *r* = 0.520, *P* = 0.023; respectively), while FCs between the left OFC and the left ventral striatum, the left OFC and the left ACC were positively correlated with VAS value (*r* = 0.751, *P* < 0.001; *r* = 0.468, *P* = 0.043; respectively) (Figures 3B–E). The mediating analysis exhibited that the oral-facial pain had a significant overall effect on depression (*P* = 0.049), while depression also had a significant overall effect on oral-facial pain (*P* = 0.039). Both relationships were completely mediated by the FC value between the left OFC and left ventral striatum. The statistical values of the specific mediation model were as follows: Path c, *P* = 0.002; Path a, *P* < 0.001; Path b, *P* = 0.042; Path c’, not significant; Standardization indirect effect = 0.481, 95% confidence interval: 0.068, 1.360. Path c, *P* = 0.002; Path a, *P* < 0.001; Path b, *P* = 0.031; Path c’, not significant; Standardization indirect effect = 0.353, 95% confidence interval: 0.072, 0.878 (Figures 3F,G).

## Discussion

This study adopted the method of functional separation and integration to explore the relationship between abnormal neural spontaneous activity and clinical symptoms in TMD patients in resting state and analyze the FC characteristics between brain regions related to emotion regulation. The results confirmed that TMD patients had localized neural activity abnormalities in regions within the limbic lobe and sensorimotor cortex, and FC abnormalities from PHG to vermis and PCC. The ALFF values of the right PHG and the FC values of the right PHG in the vermis were correlated with depression and pain, and the correlation between FC and depression was completely pain-mediated. In addition, we found that FC abnormalities in MRC of TMD patients, especially FC from the OFC to the striatal-frontal regions and DLPFC to the amygdala, were positively correlated with clinical variables. More importantly, abnormal FC in the left striatal-orbitofrontal pathway can mediate the association between pain and depressive symptoms. These altered brain regions are involved in several aspects of pain regulation, such as emotional, cognitive, and sensory functions, and maybe the underlying neuropathological basis of TMD symptoms.

### Altered Brain Activity Implicated in Regions Within the Default Mode Network in Temporomandibular Disorders Patients

The default mode network (DMN), mainly composed of mPFC, PCC, and lateral parietal cortices, plays a critical role in monitoring the environment, emotional processing, self-introspection, and episodic memory retrieval (Greicius et al., 2003; Fox et al., 2005). Individual differences in tendencies to attend to pain, with increased activity in the DMN during routine mind-wandering away from pain (Kucyi et al., 2013). Previous studies have shown functional reorganization of the DMN across chronic pain conditions (Kucyi et al., 2014; Zhang et al., 2018; Jones et al., 2020). Kucyi et al. (2014) reported that TMD patients had abnormal FC within DMN and between the DMN and pain-related networks, which may be the mechanism of the degree of reflection on chronic pain. As a key structure in the limbic system, PHG is also often reported as a part of DMN, which is involved in memory, cognition, and emotion regulation. The mood-related medial temporal region, including the hippocampus, PHG, and temporal pole, has been reported to be easily activated by painful muscle stimulation (Takahashi et al., 2011). Nascimento et al. (2019) found reduced availability of μ-opioid receptors in PHG during persistent masseter pain in patients with myofascial TMD. In addition, increased baseline BOLD signal variability in the medial temporal region in this type of patient may be associated with adverse emotional responses to muscle pain stressors (Lim et al., 2021). Exacerbation of pain by negative emotions is associated with abnormal activity in the hippocampal formation (Ploghaus et al., 2001). Our study showed increased ALFF values in the PHG and enhanced FC between PHG and PCC in TMD patients, and partly positively correlated with pain and depressive symptoms. These results further confirm that dysfunction of the DMN is associated with pain-related negative emotional thinking patterns in TMD patients. In particular, the PHG may be a more characteristic brain region.

### Altered Brain Activity Implicated in Regions Within the Cerebellar-Related Network in Temporomandibular Disorders Patients

The cerebellum had anatomical and functional connections to multiple regions of the frontal cortex and limbic region (Baumann et al., 2015). Mounting evidence has implicated that the cerebellum was associated with all dimensions of pain, including emotion, cognition, sensory function, and motor control (Allen et al., 2005; Moulton et al., 2010). Increased activity in the cerebellum (both lateral and vermis) may be due to active inhibition of Purkinje cells in a negative feedback loop to facilitate nociception and appropriate cerebellar output (Saab and Willis, 2001). In addition, cerebellum activation may also be related to the transmission of negative emotional states caused by pain and mental stress (Wittbrodt et al., 2020). RS-fMRI studies have shown abnormal local brain activity in the cerebellum and increased FC between the cerebellum and hippocampus in patients with chronic pain, which are closely related to pain perception and emotion regulation (Wang et al., 2015, 2016; Wei et al., 2020; Gui et al., 2021). In this study, TMD patients showed decreased ALFF of the right cerebellum_crus2 and increased FC between the PHG and vermis, and associated with the degree of oral-facial pain and depressive symptoms. By applying mediation analysis, we found that the relationship between FC of right PHG with vermis and psychological symptoms was entirely mediated by pain.

### Altered Brain Activity Implicated in Regions Within the Sensorimotor Network in Temporomandibular Disorders Patients

The precentral gyrus and SMA are components of sensorimotor networks and pain intensity pathways involved in response selection, sensory information generation, and discriminating properties of pain (Oshiro et al., 2009). Previous studies have consistently exhibited functional abnormalities in sensorimotor networks in patients with chronic pain (Wang et al., 2015; Zhang et al., 2017; Qin et al., 2020; Chen et al., 2021). He et al. (2014) reported abnormal fALFF in the precentral gyrus and SMA in TMD patients and correlated with the vertical centric relation-maximum intercuspation discrepancy. Increased neural activity in the precentral gyrus can reflect sensory pain responses to recurrent trigeminal neuralgia, motor inhibition of the maxilla, and facial muscle tension (Ellingson et al., 2012; Wang et al., 2015). The SMA is not only associated with pain anticipation but also plays a critical role in integrating emotional and cognitive functions (Nachev et al., 2008; Oshiro et al., 2009). TMD patients showed increased task-induced responses in the supplementary and primary motor areas during the cognitive interference tasks (Weissman-Fogel et al., 2011). Recruitment of these motor areas may be a compensatory mechanism to meet increased demands for motor planning and performance. This study found that TMD patients had increased ALFF in the precentral gyrus and SMA, which may be closely related to the long-term pain state and hypersensitivity to pain in these patients, and affect emotional and cognitive functions to a certain extent.

### Altered Functional Connectivity Within Mood-Regulating Circuits in Temporomandibular Disorders Patients

We found that TMD patients had abnormal FC between the selected regions within MRC, and the regions involved also belong to the fronto-striatal-limbic circuits, which are considered to support emotion processing and regulation, reward processing, and cognitive control.

There are extensive structural connections among the OFC, the striatum, and the limbic regions (such as the ACC), which are major dopaminergic projection regions and vital components of the reward system (LeDoux and Brown, 2017). Dopaminergic pathways are thought to be implicated in the pathophysiology of TMD. The level of plasma dopamine was positively correlated with pain intensity and perceived mental stress in TMD patients (Christidis et al., 2014; Dawson et al., 2016). The experience of pain relief can be regarded as a reward for chronic pain patients, so the role of the reward system permeates the progression of TMD (Lin, 2014). The OFC is an expectation region of value and reward for pain and is involved in pain processing and regulation (Fumal et al., 2006; Lin, 2014; Ushio et al., 2020). Previous studies have shown abnormalities in the structure and pain-induced activation of the OFC in TMD patients, some of which are related to pain and hypersensitivity (Moayedi et al., 2011, 2012). The ventral striatum and ACC are associated with the affective-motivational component of pain and can influence goal-directed behavior independently of reward (Haber and Knutson, 2010; Nakata et al., 2014). Indeed, the ACC and striatum play critical roles in pain control in TMD patients (Gerstner et al., 2012; Ichesco et al., 2012; He et al., 2018). Our study found that FC was enhanced between bilateral OFC, between OFC and ventral striatum, and between OFC and ACC. Correlation analysis showed that some FC abnormalities were positively correlated with the pain degree and negative emotions of TMD patients. These results are consistent with increased FC in reward circuits in other types of chronic pain (Ushio et al., 2020; Zhang et al., 2022). Enhanced FC within the dopaminergic regions of the reward network in TMD patients may compensate for the downregulation of the descending pain system, participating in pain perception by increasing emotional experience and creating predictions. In particular, we also found that abnormal FC in the left striatal-orbitofrontal pathway can mediate the association between pain and depressive symptoms, which may be the neural mechanism underlying the interaction between pain and negative emotions in TMD patients.

As the core component of the central executive network, the DLPFC plays a regulatory role in pain perception and emotional processing through the descending inhibitory pathway (Lorenz et al., 2003). Lim et al. (2021) reported that decreased BOLD signal in the right DLPFC is associated with the cumulative number of TMD pain days over six months. The amygdala is a complex and deep brain structure in MRC and is involved in emotional processing. Neuroimaging studies reported that amygdala dysfunction is associated with chronic pain and psychiatric disorders (Simons et al., 2014; Wang et al., 2015). The inhibitory effect of DLPFC on negative emotions is proven to be through its connection with the amygdala (Davidson et al., 2000; Lorenz et al., 2003). Disruption of the DLPFC-amygdala circuitry may implicate a lack of inhibitory control of nociceptive input in chronic pain patients (Zhang et al., 2018). In line with these findings, the present study found that the FC between DLPFC and amygdala decreased in TMD patients. We speculate that the disconnection of this descending inhibitory pathway is one of the vital factors for persistent pain and related mood disorders in TMD patients.

Several limitations should be noted in the present study. First, the sample size was relatively small, and the effects of pain laterality and mood disorders on brain function should be further analyzed by grouping. Second, longitudinal observation was lacking, so the relationship between the changes of FC and alters in the emotional state could not be determined. Third, this study did not include assessments of cognitive function and other mental states. Future studies should explore the relationship between cognitive function and mental disorders in TMD patients. Finally, fMRI studies under emotion-related tasks are needed to explore abnormal activation of brain regions under emotional stress.

## Conclusion

In summary, the present study adopted functional separation and integration analysis to show that TMD patients had aberrant spontaneous activity and FC in the DMN, sensorimotor network, and pain-related regions, as well as dysfunction of the fronto-striatal-limbic circuits. In addition, this study provides further neurobiological evidence that the occurrence and development of TMD negative emotions may be related to the dysfunction of dopamine pathway components (especially hippocampus complex, OFC, striatum) induced by chronic pain. Therefore, the abnormal dopaminergic FC pattern linking oral-facial pain and depression may serve as indicators of underlying psychiatric and pathophysiological neural mechanisms. Abnormal FC patterns may also guide the personalized pain treatment of TMD comorbidity with negative emotion, such as the application of serotonin reuptake inhibitor drugs.

## Data Availability Statement

The raw data supporting the conclusions of this article will be made available by the authors, without undue reservation.

## Ethics Statement

The studies involving human participants were reviewed and approved by the Ethics Committee of The Affiliated Hospital of Hangzhou Normal University. The patients/participants provided their written informed consent to participate in this study.

## Author Contributions

X-FC, PH, and JL designed and supervised the project. PH, K-HX, YC, BW, XH, and LQ collected the data. X-FC, K-HX, Y-HJ, M-WW, and JL processed and analyzed the data. All authors revised the manuscript and approved the final version.

## Conflict of Interest

The authors declare that the research was conducted in the absence of any commercial or financial relationships that could be construed as a potential conflict of interest. The reviewer RH declared a past co-authorship with one of the author JL to the handling editor.

## Publisher’s Note

All claims expressed in this article are solely those of the authors and do not necessarily represent those of their affiliated organizations, or those of the publisher, the editors and the reviewers. Any product that may be evaluated in this article, or claim that may be made by its manufacturer, is not guaranteed or endorsed by the publisher.

## Footnotes

1
http://cn.mathworks.com/products/matlab/

2
http://www.restfmri.net/forum

3
http://www.fil.ion.ucl.ac.uk/SPM/

4
http://lcni.uoregon.edu/~jolinda/MRIConvert/
